# The complete chloroplast genome sequence of *Polygonum chinense* L.

**DOI:** 10.1080/23802359.2019.1693931

**Published:** 2020-05-22

**Authors:** Benxia Yu, Jiang Liu, Xiang Liu, Xiaozhong Lan, Xianyou Qu

**Affiliations:** a Chongqing Institute of Traditional Chinese Medicine, Chongqing, China; b Tibetan Collaborative Innovation Centre of Agricultural and Animal Husbandry Resources, Food Science College, Tibet Agriculture & Animal Husbandry University, Nyingchi of Tibet, China

**Keywords:** *Polygonum chinense* L., complete chloroplast genome, phylogenetic analysis

## Abstract

*Polygonum chinense* is a traditional natural plant pharmaceutical with antimicrobial, antioxidant, and antidiarrheal effects and mainly distributed in China and Southeast Asian countries. The complete chloroplast sequence of *P. chinense* has been determined in this study. The cpDNA was 158,981 bp in length, containing a pair of inverted repeats of 30,872 bp each separated by a large and small single-copy region of 84,347 and 12,890 bp, respectively. The genome contained 86 protein-coding genes, eight rRNA genes, and 37 tRNA genes. The overall GC content of the chloroplast genome was 38%. Phylogenetic tree demonstrated that *P. chinense* closely related to *Rheum palmatum* and *Rheum wittrockii.*

*Polygonum chinense* (*P. chinense*) Linn. is a common medicinal plant distributed in China and Southeast Asian countries. *Polygonum chinense* belongs to the *Polygonum* genus, Polygonaceae family. The rhizomes of the drug are medicinal part and used to clear away heat and detoxification, and dissipate swelling (Anren, 1998). As a traditional natural plant pharmaceutical, *P. chinense* has been used for skin infectious diseases and healing inflammatory wounds or insect stings and snakebites (Srividya et al. 2012). Ethanol extract and isolated bioactive substances exerted antidiarrheal effects (Hai-Tao et al. 2013). In this study, we assembled and characterized the whole chloroplast genome of *P. chinense* and learned more about genetic information of this species, which can contribute to the conservation, and provide useful help for population genetics studies of *P. chinense*. The annotated genome sequence has been deposited into Genbank under the accession number MN627221.

The plant materials of *P. chinense* were collected from Nyingchi (Tibet, China; N: 30°01′32.50″, E: 95°00′49.16″). The specimens of *P. chinense* have been kept in Tibet Agriculture & Animal Husbandry University and specimen Accession number is 542621150719690LY. The modified CTAB method (Allen et al. 2006) was used to extract the total genomic DNA of *P. chinense.* The *P. chinense* DNA sample was sequenced as the paired end using the Illumina HiSeq 2000 platform (Illumina, San Diego, CA). About 2.5 G base pairs of sequencing data in total were obtained and then de novo assembled using CLC genome assembler program (ver.4.06 beta, CLC Inc, Aarhus, Denmark) as previously described (Kim et al. 2015). The sequenced fragments were assembled and corrected using Geneious Prime 2019.2.1 (Kearse et al. 2012), with *Rheum palmatum* (GenBank: NC027728) as the reference. DOGMA program was used for annotation of the complete chloroplast genome (Wyman et al. 2004).

The complete chloroplast genome of *P. chinense* has a circular molecular structure of 158,981 bp in length with 38% GC content, consisting of a large single-copy (LSC) region of 84,347 bp and a small single-copy (SSC) region of 12,890 bp, separated by a pair of 30,872 bp IR region. We annotated 131 genes, including 86 protein-coding genes, 37 tRNA genes, and 8 rRNA genes.

To investigate the phylogenetic relationship of *P. chinense*, the phylogenetic tree was generated by the neighbour-joining (NJ) method from alignments created by the MAFFT (Katoh and Standley 2013) and selected 10 Caryophyllales species published complete chloroplast genomes from GenBank to assess the genetic and phylogenetic relationship with *P. chinense* (Figure 1). Phylogenetic analysis showed that *P. chinense* is most closely related to *Rheum palmatum* and *Rheum wittrockii.* The complete chloroplast genome sequence of *P. chinense* can be utilized for DNA barcoding and phylogenomic studies of Polygonaceae.

**Figure 1.**
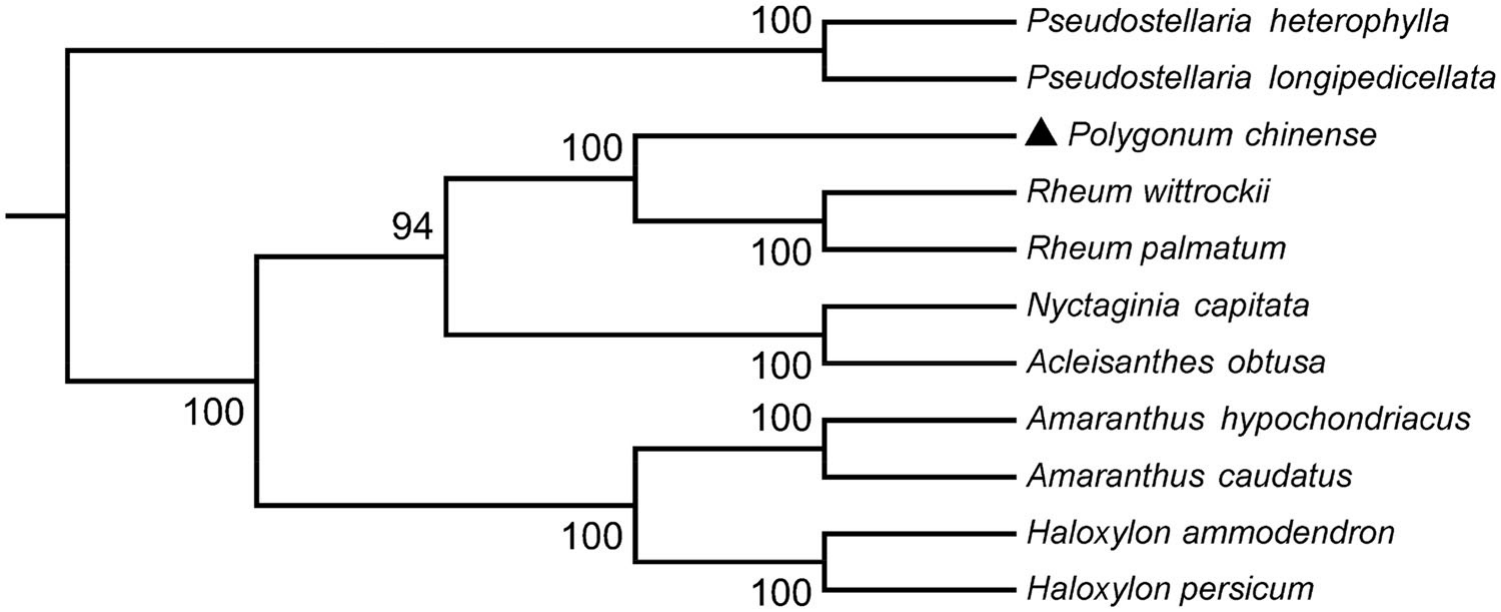
Phylogenetic tree based on the complete chloroplast genome sequences of *P. chinense* and 10 other species. The tree was generated using a NJ method by MEGA7 with 1000 bootstrap replicates. Numbers on the nodes indicate bootstrap values. The chloroplast genome sequences used to construct the phylogenetic tree are *Pseudostellaria heterophylla*(MK801111), *Pseudostellaria longipedicellata* (MH373593), *Rheum wittrockii* (KY985269), *Rheum palmatum* (NC027728), *Nyctaginia capitate* (MH286318), *Acleisanthes obtuse* (MH286321), *Amaranthus hypochondriacus* (KX279888), *Amaranthus caudatus* (MG836508), *Haloxylon ammodendron* (KF534478), and *Haloxylon persicum* (KF534479).
